# Malaria from hyperendemicity to elimination along international borders in Yunnan, China during 2003‒2020: a case study

**DOI:** 10.1186/s40249-022-00972-2

**Published:** 2022-05-10

**Authors:** Hui Liu, Yaowu Zhou, Yan Deng, Zurui Lin, Canglin Zhang, Qiyan Chen, Chun Wei, Kaixia Duan, Peng Tian, Hongning Zhou, Jianwei Xu

**Affiliations:** Yunnan Institute of Parasitic Diseases, Yunnan Provincial Centre of Malaria Research, Yunnan Provincial Key Laboratory of Vector-Borne Diseases Control and Research, Yunnan Institute of Parasitic Diseases Innovative Team of Key Techniques for Vector Borne Disease Control and Prevention, Training Base of International Scientific Exchange and Education in Tropical Diseases for South and Southeast Asia, Puer, 665000 China

**Keywords:** Malaria, Control, Elimination, Border area, International collaboration, Yunnan, China

## Abstract

**Background:**

Border malaria is one of the most intractable problems hindering malaria elimination worldwide. Movement of both the human population and anopheline mosquitoes infected with *Plasmodium* spp. can cause cross-border malaria transmission. The Yunnan border area was still hyperendemic for malaria in the early part of this century. The objective of this case study was to analyze the strategies, interventions and impacts of malaria control and elimination in the Yunnan border area.

**Main text:**

A total of 10,349 malaria cases and 17.1 per 10,000 person-years of annual parasite incidence (API) were reported in the border area in 2003. Based on natural village-based stratification, integrated interventions, including mass drug administration for radical cures and preventive treatment, clinically presumptive treatment of all febrile patients for malaria and indoor residual spraying or dipping bed nets with insecticides were successfully carried out from 2003 to 2013. The overall API was reduced to 0.6 per 10,000 person-years by 2013, while effective cross-border collaboration interventions dramatically reduced the malaria burden in the neighbouring border areas of Myanmar. From 2014 forward, the comprehensive strategy, including universal coverage of surveillance to detect malaria cases, a rapid response to possible malaria cases and effective border collaboration with neighbouring areas, successfully eliminated malaria and prevented reintroduction of malaria transmission in the Yunnan border area.

**Conclusions:**

In Yunnan malaria burden has successfully reduced by dynamically accurate stratification and comprehensive interventions; and then the region achieved elimination and prevented reintroduction of malaria transmission through intensive surveillance, rapid response and border collaboration. Other border areas should perform their own intervention trials to develop their own effective strategy.

**Graphical Abstract:**

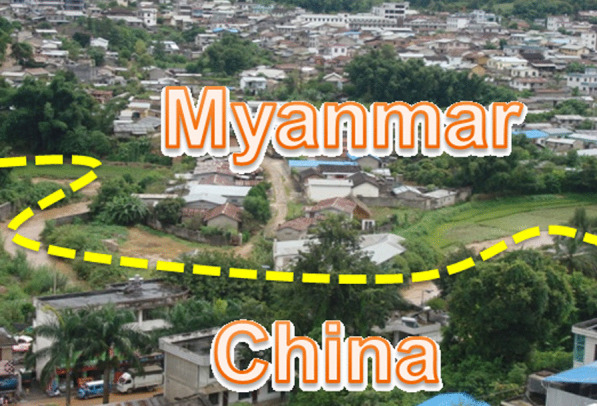

**Supplementary Information:**

The online version contains supplementary material available at 10.1186/s40249-022-00972-2.

## Background

The World Health Organization (WHO) certified China malaria-free status on June 30, 2021 [1, 2]. Yunnan Province in southwestern China shares 4060 km of border with Myanmar (1997 km), Laos (710 km) and Vietnam (1353 km). Yunnan is a unique province with malaria ecology and vector system similar to those of five other countries in the Great Mekong Sub region (GMS) [3]. Frequent migrants and anopheline mosquitoes infected with *Plasmodium* spp. crossing the border, and underdeveloped health services can lead to cross-border transmission of malaria parasites [4, 5]. These factors underline the original hyperendemicity in the Yunnan border area [6], and malaria elimination was truly difficult in the area. Malaria in the Yunnan border area definitely impaired its elimination in China [7]. Malaria elimination in China is a remarkable achievement and the culmination of seven decades of dedicated effort by the national malaria programme and its partners. Border malaria elimination in Yunnan has strongly contributed to this remarkable achievement [8]. Currently, Malaria is a continuous public health problem worldwide. Due to health service disruptions during the coronavirus disease 2019 (COVID-19) pandemic, there were an estimated 241 million malaria cases in 2020, increased from 227 million in 2019, and malaria deaths increased by 12% compared with 2019, to an estimated 627 thousand [9]. Border collaboration has promoted malaria elimination in the Yunnan border area [10]. Under the context of the COVID-19 pandemic, border collaboration for malaria control activities is limited when border crossings are strictly limited. The surveillance data of the cross-border joint prevention and control project of malaria and dengue fever in Yunnan of China and GMS showed malaria resurgence in part of the border area of neighboring countries. For example, the Laiza and nearby areas in Kachin Special Region II (KR2) of Myanmar reported 274 malaria cases in 2019 followed by 1587 cases in 2020. The resurgence of malaria in some border areas of neighboring countries suggests that China should prepare well to respond to the reintroduction of malaria transmission in the Yunnan border area for the post COVID-19 era. The objective of this case study was (1) to analyze the strategies and interventions used from malaria control to its elimination and their impact during 2003‒2020, and (2) to present a strategy of preventing the reintroduction of malaria transmission in the Yunnan border area.

## Methods

### Study site

The border county is defined as the border area in this study. There are 25 border counties in Yunnan, namely 17 counties bordering Myanmar, one (Mengla) with Myanmar and Laos, one (Jiangcheng) with Laos and Vietnam, and six counties with Vietnam. The Yunnan border area has a tropical or subtropical monsoon climate and is populated by 9,093,082 people in 2020. A hot climate, adequate precipitation and forests provide a suitable environment for the growth and reproduction of mosquitoes and for malaria transmission. With a complex vector community, *Anopheles minimus* and *An. sinensis* were identified as the primary and secondary vectors of malaria in this area [11, 12]. Year-round malaria transmission occurred in most parts of the border area prior to elimination. All four of the parasite species (i.e., *P. falciparum, P. vivax, P. malariae* and *P. ovale*) were detected in the area [13]. There were no natural or artificial barriers along the boundary prior to the COVID-19 pandemic. Thirteen indigenous ethnic minorities live across the boundary. The border area is an underdeveloped area with poor communities, marginalized populations and weak health services. The border areas of the three neighboring countries present civil unrest (mainly in Myanmar), unpermitted border crossers and a high malaria burden [14]. Each of these factors challenged the feasibility of border malaria elimination in the Yunnan border area.

### Data sources and collection

To collect data on malaria cases, intervention activities and control strategies, all available paper-based records related to border malaria surveillance and interventions from 2003 to 2020 were reviewed at the Yunnan Institute of Parasitic Diseases (YIPD). As the Chinese Information System for Disease Control and Prevention (CISDCP) began to cover all Yunnan’s counties since 2008 [15, 16]; therefore, the relative data during 2008–2020 were obtained from the CISDCP. In addition, all available documents and literature about the border malaria situation and control activities in Yunnan and neighboring countries (Vietnam, Laos and Myanmar) were also reviewed. These studies and documents include original work records, books, annals, guidelines and operational manuals about malaria control and elimination in Yunnan.

### Data analysis

**Fig. 1:**
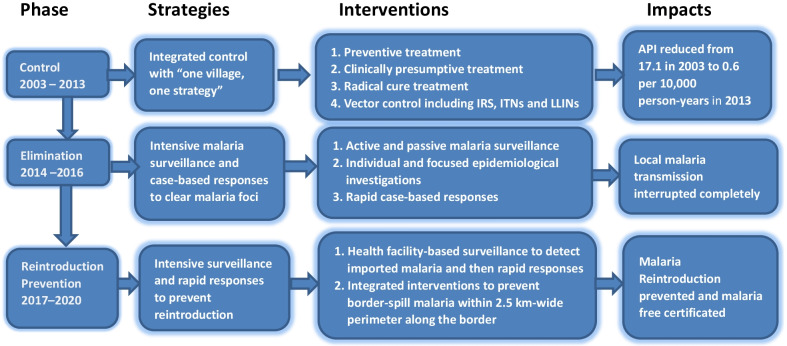
The malaria intervention flow from hyperendemicity to elimination in the Yunnan border area. *API* annual parasite incidence, *IRS* indoor residual spraying with insecticides, *ITNs* insecticide-treated bed nets, *LLINS* long lasting insecticidal bed nets

To analyse and present the data, the malaria programme from hyperendemicity to elimination in the border area during 2003‒2020 was divided into three phases, namely, control phase (2003‒2013), elimination phase (2014‒2016) and reintroduction prevention phase (2017‒2020) (Fig. 1). This phase division was based on the WHO’s recommendation on malaria programme phases and milestones on the path to malaria elimination [17] and the local context in Yunnan Province. The control phase was the period with an overall annual parasite incidence (API) ≥ 1.0 per 10,000 person-years, the elimination phase was the period with API < 1.0 per 10,000 person-years but with indigenous malaria cases, and the reintroduction prevention phase was the period from local interruption of malaria transmission forward.

The key events that were considered having significant impact on malaria control and elimination in the Yunnan border area were summarized to list in Table 1. For each phase, the strategies and interventions were described, including stratification of malaria areas, treatment of malaria cases, vector control, surveillance and focus responses from malaria hyperendemicity to elimination. To present the malaria case surveillance and drug-based prevention, annual coverage of laboratory tests for malaria and preventive treatment were calculated for each year of the three phases.

**Table 1:** The key events of malaria control and elimination in the Yunnan border area, China, 2003‒2020

Year	Key events
Control phase	
2003	In January, the first round of the China’s Global Fund to Fight AIDS, Tuberculosis and Malaria (GFATM) malaria program was rolled out in 25 border counties
2005	On June 7, the first collaborative document of cross border malaria control was signed between China and Myanmar. “The joint malaria control project along China–Myanmar Border” regularly exchanged information and conducted activities of malaria control since 2005 [20, 21]
2007	In July, the sixth round of the China’s GFATM malaria program on cross border malaria control was launched in China’s 12 border counties and Myanmar’s four special regions [15]
2010	On July 1, the national malaria elimination action was launched in China including Yunnan’s 25 border counties [25]
2012	On January 1, the tenth round of the China’s GFATM malaria program on cross border malaria control was rolled out in China’s seven border counties and Myanmar’s five special regions [15]
2013	The tenth round of the China’s GFATM malaria program stopped in China on December 31 [15]
Elimination phase	
2014	The second phase of the tenth round of the China’s GFATM malaria program was consolidated into the Myanmar’s GFATM project since January 1 [15]
2014‒2016	There was a slight resurgence of malaria incidence in Myanmar’s Kachin Special Region II (KR2) and Shan Special Region II (Wa State) that led to an increase of imported malaria cases in Yunnan [18, 35]
2014	China and Myanmar collaboratively controlled the outbreak of *Plasmodium falciparum* in Wa State and prevented malaria importation into China [35]
2014	The impact evaluation of cross China–Myanmar border malaria control program during 2007‒2013 was carried out. Results indicated that the malaria burden was reduced by 95% in China’s 19 border counties based on the API and by 90% in Myanmar’s five special regions based on the malaria parasite prevalence [15]
2016	The last indigenous malaria case of China was reported from Yingjiang county on China–Myanmar border on April 17, 2016 [27]
Reintroduction prevention phase	
2017‒2019	China and Myanmar collaboratively controlled the resurgence of malaria incidence in Laiza and nearby areas, KR2, Myanmar. The number of malaria cases was reduced from 2080 cases in 2016 to 274 cases in 2019 in the Laiza and nearby area
2018	In March, the Yunnan health and Family Planning Commission released “The notification on further standardizing malaria elimination work and process” to clear the responsibility of general health service in malaria surveillance [10]
2019	In January, the “3 + 1” strategy for border malaria elimination and preventing reintroduction of malaria transmission was developed and formulated [10]
2020	In January, Yunnan passed the national technical assessment of malaria elimination. In June, Yunnan passed the finally national assessment of malaria elimination

Drug-based treatment is the primary intervention to clear malaria parasite reservoirs and interrupt transmission [18]. To solve the challenges of asymptomatic and submicroscopic parasite density (especially for *P. vivax*), and the limitations of microscopist ability and rapid diagnostic tests (RDTs), an expanded treatment strategy was used during the control phase. Ratios of the number of laboratory-confirmed malaria cases versus the number of people treated with antimalarial drugs were calculated.

To present the impact of these strategies and interventions, the API of the overall border area was calculated for each year of 2003–2013. When local transmission was interrupted, malaria was mainly imported from endemic areas of other countries, and calculation of the API was not appropriate [17]. Only the number of imported malaria cases detected and their infection sources were counted since 2014. The years of local certification of malaria free for eight border prefectures were used to document the impact of elimination interventions.

## Results

### Control phase from 2003 to 2013

#### Integrated control strategies of “one village, one strategy”

Facing hyperendemicity in this early century, the approach of “one village, one strategy” that was developed and started in Yunnan in the early 1990s, and continuously carried out during 2003‒2013. This strategy categorized all natural villages into four types each year dynamically according to their malaria incidence in the last 3 years. Type I was villages with API ≥ 1%, or malaria clinical attack rate (proportion of people who had clinical symptoms of malaria among all residents in the village) in last year ≥ 10%; Type II was villages with API < 1%, or malaria clinical attack rate < 10% in last year, but with indigenous cases in the last 3 years; Type III was villages without indigenous cases, only with imported cases in the last 3 years; and Type IV was villages without any malaria cases in the last 3 years (Additional file 1: Table S1) [19].

#### Border collaboration and funding application

Cross border collaboration was initiated to reduce malaria burden in the border areas of neighbouring countries during this phase. The former Ministry of Health of China and the Ministry of Health and Sports of Myanmar signed “The Agreement of Cross Border Malaria Control” on June 7, 2005 [20]. “The joint malaria control project along the China–Myanmar Border” has been carried out since 2005 [21]. Under the agreement framework, YIPD and Health Poverty Action successfully applied for and carried out the sixth and tenth rounds of the Global Fund to Fight AIDS, Tuberculosis and Malaria (GFATM) with two malaria projects conducted along the China–Myanmar border from 2007 to 2013 [15].

#### Natural village-based stratification and interventions

**Fig. 2:**
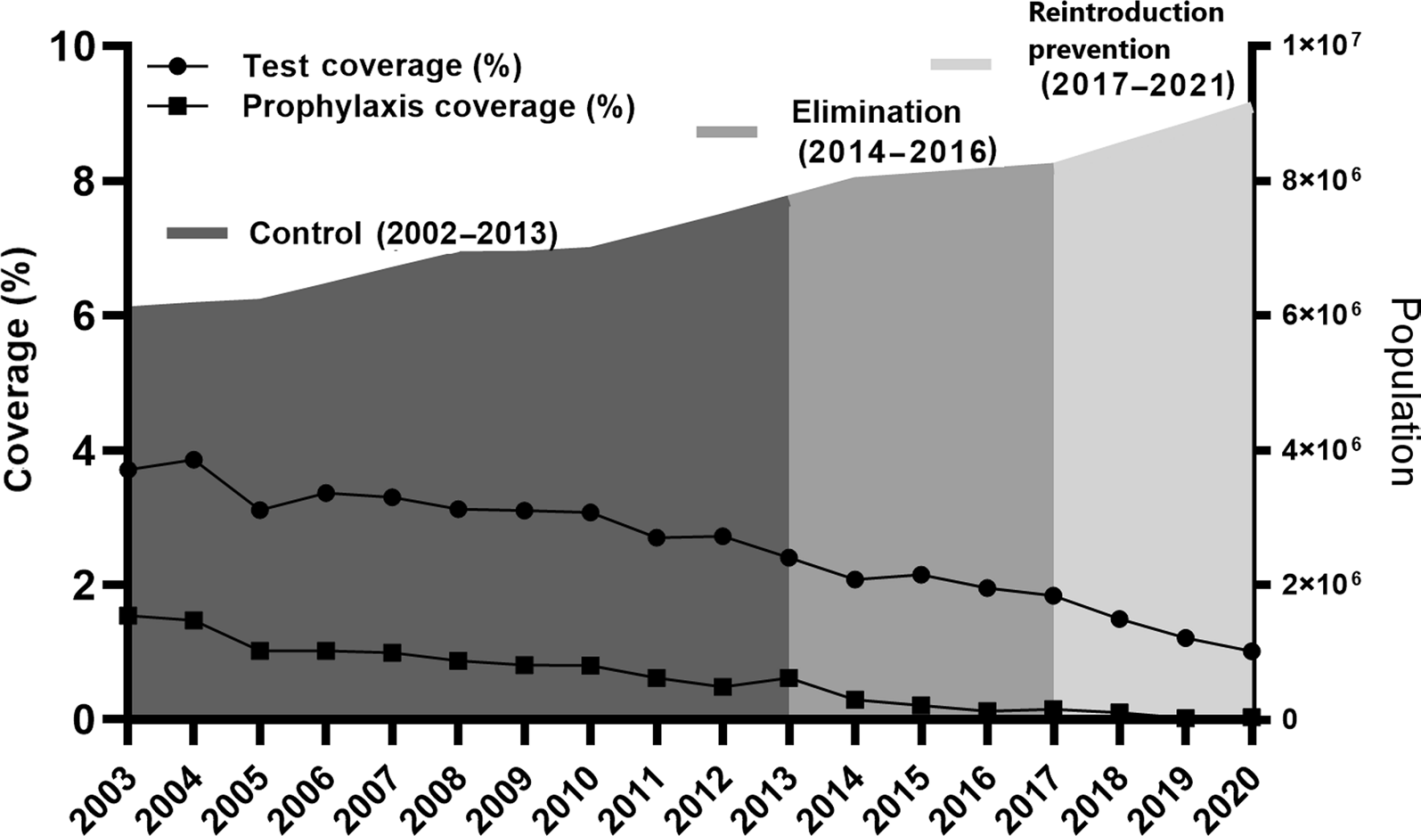
The annual coverage of laboratory tests for malaria parasites and preventive treatment in the Yunnan border area, 2003‒2020

To solve the problems of high morbidity, specificity and complexity, the strategy of natural village-based stratification and interventions was continuously conducted in the border area. Mass drug administration for radical cure treatment was conducted in type I villages in the low transmission season (December–February of next year) and for preventive treatment in the high transmission season (May‒October). Radical cure treatment was only administered to people with a malaria attack history in the last 2 years in type II‒IV villages [19, 22]. With a decreasing malaria incidence, Fig. 2 indicates that the coverage of preventive treatment, namely, the percentage of people with at least one drug administration for prophylaxis, decreased from 1.5% in 2003 to 0.6% in 2013 (Additional file 1: Table S2). To accelerate the Yunnan pace of malaria elimination, radical cure treatments were expanded to clear parasite reservoirs as soon as possible. The ratio of the number of people with radical cure treatment versus the number of laboratory confirmed malaria cases increased from 3.2 in 2003 to 17.3 in 2010, followed by reduction to 5.1 in 2013 (Table 2). Meanwhile, indoor residual spraying (IRS), insecticide-treated bed nets (ITNs) with pyrethroid or delivering long lasting insecticidal bed nets were conducted in type I and II villages. IRS with pyrethroid insecticides was only carried out in houses of malaria patients and their neighbours in type III and IV villages [19].

**Table 2:** The ratios of clinically presumptive treatment (CPT) and radical cure treatment (RCT) versus laboratory confirmed malaria cases in the Yunnan border area, 2003‒2020

Phases	Year	No. confirmed cases	No. CPT	Ratio of CPT vs confirmed cases	No. RCT	Ratio of RCT vs confirmed cases
Control	2003	10,349	33,885	3.3	32,866	3.2
	2004	8515	28,022	3.3	31,811	3.7
	2005	9538	31,183	3.3	31,498	3.3
	2006	8666	27,630	3.2	29,740	3.4
	2007	4872	17,296	3.6	29,103	6.0
	2008	2762	9099	3.3	27,195	10.0
	2009	2045	6715	3.3	26,381	12.9
	2010	1570	2072	1.3	27,136	17.3
	2011	850	1159	1.4	9466	11.1
	2012	449	631	1.4	4670	10.4
Elimination	2013	475	476	1.0	2404	5.1
	2014	393	398	1.0	1742	4.4
	2015	478	506	1.1	509	1.1
	2016	318	336	1.1	488	1.5
Reintroduction prevention	2017	264	284	1.1	407	1.5
	2018	169	172	1.0	343	2.0
	2019	149	154	1.0	204	1.4
	2020	137	137	1.0	187	1.4

#### Impacts on malaria burden

**Fig. 3:**
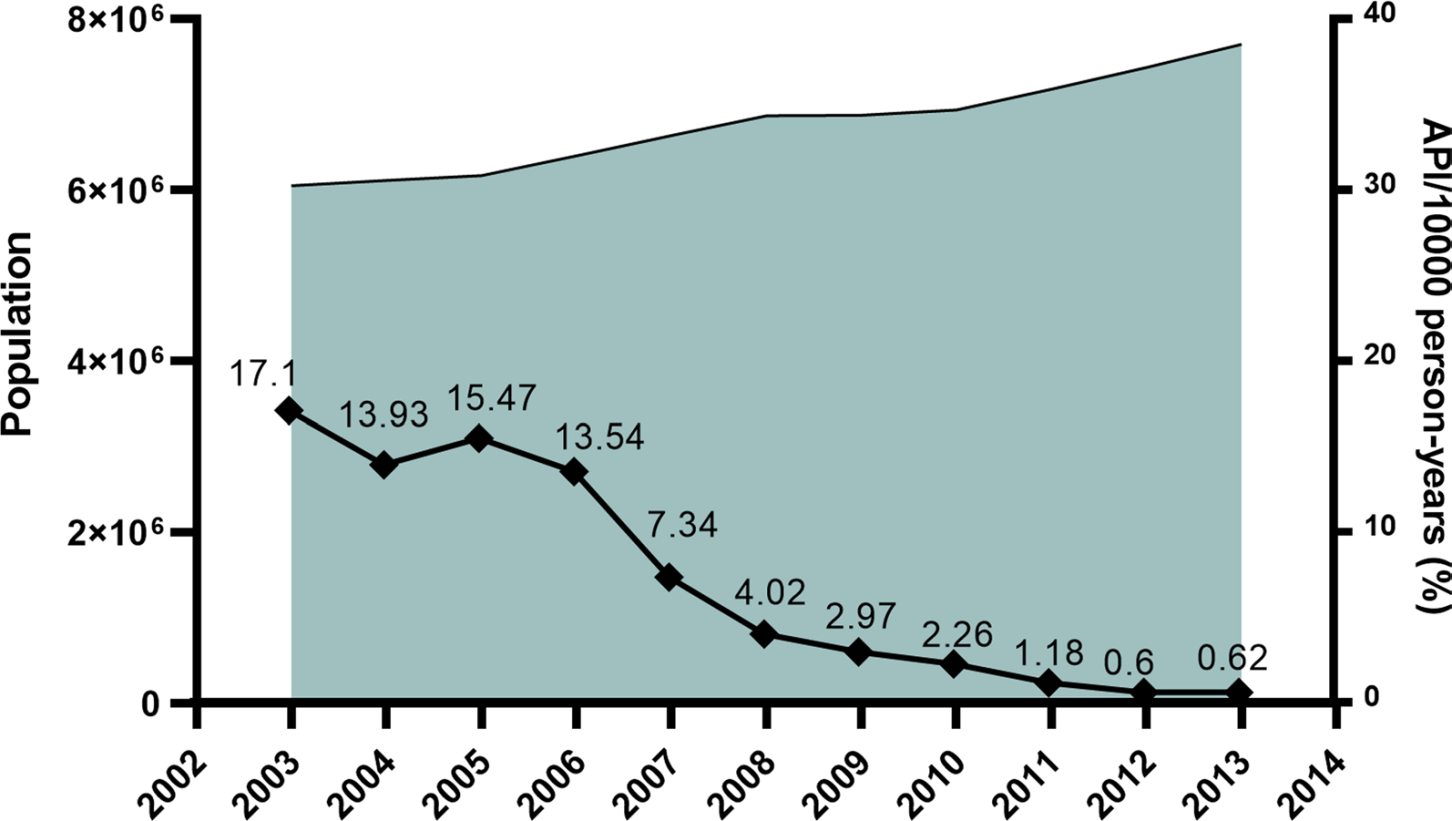
The annual parasite incidence (API) in the Yunnan border area, 2003‒2013

In 2003, a total of 10,349 cases and 17.1 per 10,000 person-years of API were reported in the Yunnan border area (Fig. 3). The number accounted for 67.1% of 15,431 confirmed malaria cases across Yunnan Province. A survey found that more than 90.0% malaria cases were underreported in the border area in 2002. This underreported rate was higher than the mean underreported rate (88.8%) in Yunnan [23]. Based on this survey of underreported malaria cases, it was estimated that there were approximately 100 thousand malaria cases in the border area in 2003. As a result of the intensive interventions, the API was successfully reduced to 13.5 per 10,000 person-years in 2006, followed by 2.3 per 10,000 person-years in 2010 and then 0.6 per 10,000 person-years in 2013 (Fig. 3). The dramatic reduction in malaria burden was also attributable to effective border collaboration for malaria control between China and Myanmar. The two GFATM projects successfully reduced the malaria burden by 90% in five Special Regions of Myanmar as well as by 95% in the Yunnan border area along the China–Myanmar border. The data on control activities and their impact on malaria burden were presented in detail in previously publised papers [10, 15]. The significant reduction in malaria cases made it possible to completely switch the malaria programme from control to elimination in Yunnan.

### Elimination phase from 2014 to 2016

#### Strategies of “clearing malaria foci, tracking infectious sources”

Yunnan’s 104 inland counties kept pace with the country to start malaria elimination action since 2010. Due to the higher endemicity in the Yunnan border area, malaria elimination action was actually launched in 2014. Malaria elimination requires a universal coverage of malaria surveillance and a rapid response to any suspected malaria foci [17]. The Chinese “1-3-7” strategy requests reporting malaria cases within 1 day, confirmation and investigation of malaria cases within 3 days, and an appropriate public health response to prevent further transmission within 7 days [24]. The WHO recommends that the elimination phase starts in a district where the first program reorientation has been achieved; and where health facility data show an API < 1 per 1000 person-years at risk, equal to less than 100 new cases per year in a district with a population of 100,000 people [17]. In China, the smallest unit for elimination is a county, and most counties have a population of over 1 million. The national malaria elimination program therefore recommended that the elimination phase was initiated after achieving an API < 1 per 10,000 person-years. The national standards of county stratification for malaria elimination categorized all counties into four tiers, namely, type I with the presence of confirmed local case (s) in the last 3 years, with at least 1 year having an API ≥ 1 per 10,000 person-years; type II with the presence of confirmed local case(s) in the last three years, with an API < 1 per 10,000 person-years; type III without any local cases for at least 3 years, only imported cases; and type IV without a history of any local cases, only imported cases [25]. Following the national stratification standards for malaria elimination, Yunnan categorized its 129 counties into three tiers (no type IV), namely, 19 type I counties with 17 border counties, 55 type II counties with eight border counties, and 55 type III counties in 2010. According to the stratification, every county took malaria elimination as one of the governmental work objectives to establish a leadership and technical steering team. The strategy of “clearing malaria foci (parasite reservoirs), tracking infectious sources” were conducted by intensive surveillances, epidemiological investigations and rapid public health responses.

#### Interventions for intensive surveillance and rapid response

Following “The Protocol of Yunnan Malaria Elimination Action Plan (2010‒2020)”, the interventions of intensive surveillance and rapid response were conducted [26]. A total of 481,772 febrile patents were tested by microscopy or RDTs for malaria in the border area from 2014 to 2016 (Fig. 2, Additional file 1: Table S2). Following the “1-3-7” work approaches, all 1240 malaria cases detected were reported within 1 day; individual epidemiological surveys were completed within 3 days; and focused epidemiological investigations and public health responses were conducted within 7 days [24]. Strengthened malaria surveillance ensured the timely detection of parasite infections and rapid responses to clear parasite reservoirs for preventing further transmission.

#### Impacts on malaria transmission

**Fig. 4:**
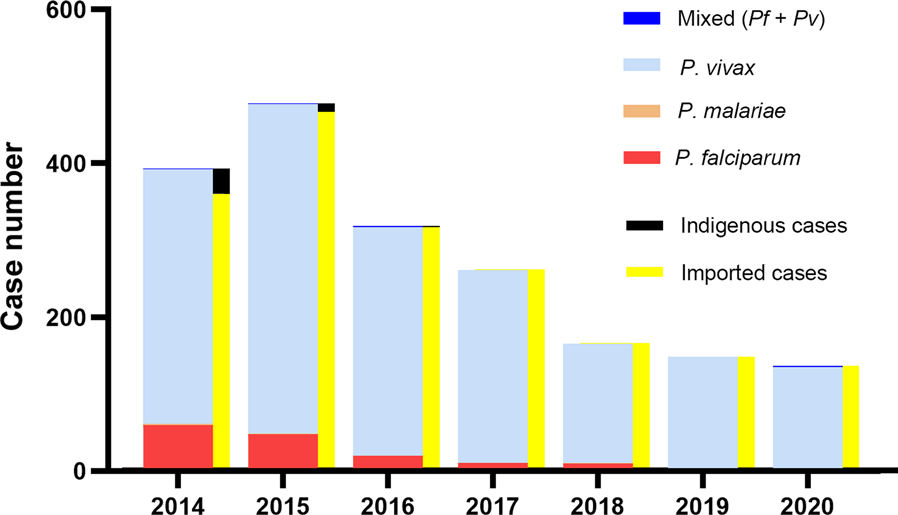
The number of malaria cases detected and the categories in the Yunnan border area, 2014‒2020. The last indigenous case (*P. vivax*) occurred in Yingjiang County on the China–Myanmar border in April 2016. It is also the last indigenous case in China

The Action Plan of China Malaria Elimination 2010–2020 scheduled reducing the API to less than 1 per 10,000 person-years in each county of the Yunnan border by the end of 2015. This goal was actually achieved by 2013 with a mean API of 0.6 per 10,000 person-years (Fig. 3), except Tengchong with an API of 2.0 per 10,000 person-years due to imported malaria cases being included in the API and imported cases accounting for more than 95% of the total cases in Tengchong County (Additional file 1: Table S5). The WHO guidelines for malaria elimination do not recommend the inclusion of imported malaria cases in the calculation of API [17]. At last, the transmission of falciparum malaria has successfully been interrupted since the last locally falciparum malaria case was reported from Cangyuan County in May 2015, and then vivax malaria transmission has finally been interrupted since the last locally vivax malaria case was reported from Yingjiang County on April 17, 2016 (Fig. 4) [27].

### Reintroduction prevention phase from 2017 to 2020

#### Strategies of timely malaria detection and response

The WHO malaria elimination certification standard is that the chain of indigenous malaria transmission by *Anopheles* mosquitoes has been interrupted nationwide for at least the past 3 consecutive years, and a country must also demonstrate the capacity to prevent reintroduction [28]. However, Yunnan borders three malaria endemic countries. Imported malaria can be caused by both border crossers and parasite-infected *Anopheles* mosquitoes, which fly over the boundary from endemic areas of neighbouring countries [10]. After the interruption of malaria transmission, the national stratification standards of malaria elimination were no longer for the actual situation in the Yunnan border area. To effectively prevent the reintroduction of malaria transmission, Yunnan further categorized 25 border counties into three tiers (types A, B and C) based on the malaria hyperendemicity in border area of neighbouring countries and the specificity of 25 border counties in 2017. The type A and B counties are to border with Myanmar or/and Laos. The type A counties are with 10 and more imported malaria cases from the border areas of neighbouring countries or with malaria cases lacking of travel history in the endemic areas of other countries during 2015‒2016. The type B counties are with less than 10 imported malaria cases from the border area of neighboring countries and the imported malaria cases with clear travel history in the endemic areas of other countries during 2015‒2016. The type C counties are only to border with Vietnam. The 12 type A counties needed more input of human and financial resources to carry out more intensive interventions, including vector control. Seven type B counties needed to strengthen malaria case surveillance. The six type C counties bordering Vietnam did not need additional investment or special interventions. The results of 291 *Anopheles* mosquito mark-release-recapture experiments in 143 localities around the world estimated that the mean distance travelled of female *Anopheles* was not more than 2.5 km [29]. An assessment of the receptivity and vulnerability was conducted for each community within 2.5 km-wide perimeter border areas of Myanmar along the boundary. The assessment result proposed a total of 16 natural villages in the threat of border-spill malaria in 2018 (Additional file 1: Table S7). Border-spill malaria is defined as a kind of imported malaria that is caused by parasite-infected *Anopheles* from the border endemic areas of neighbouring countries.

#### Health facility-based surveillance and border-spill malaria prevention

For each of the border counties, passive detection was consolidated into normal health service. Health services personnel were trained to remain vigilant to ensure universal coverage of malaria detection and react promptly to any suspected malaria cases. The unpermitted travellers cross borders frequently and present in frontier townships. With assistance from villager leaders and health workers to monitor cross border travellers, and refer febrile patients to the township hospitals for malaria test, community-based malaria detection and screening of migrants and travellers were carried out in frontier townships. To prevent the border-spill malaria, integrated interventions that include proactive and passive detection of the malaria parasites, enhancement and optimization of vector surveillance, further strengthening of timely detection with high-quality confirmed diagnosis and prompt action based on the surveillance results were carried out in these 16 high-risk reintroduction villages [10]. These interventions ensured universal coverage of malaria surveillance to detect malaria cases and timely public health responses in the Yunnan border area.

#### Impacts on malaria free certification

The threat from Vietnam and Lao PDR is slight. The overall incidence of malaria is low in Vietnam, with malaria transmission being interrupted in northern Vietnam [30]. Malaria control has also made rapid progress toward localized elimination goals in the northern provinces of Laos [31]. Yunnan first achieved malaria free status for at least 3 years in Honghe Prefecture with three counties bordering Vietnam only in 2015 [16], and then Wenshan Prefecture, with three counties bordering Vietnam in 2016. Beginning from the Honghe Prefecture, the eight border prefectures and their 25 border counties were gradually evaluated and certified for malaria free by Yunnan itself following the national standards of malaria elimination assessment (Table 3). The intensive interventions effectively prevented the reintroduction of malaria transmission to ensure timely national and WHO malaria-free certification. The China National Health Commission finally assessed and certificated Yunnan malaria free in June 2020. Experts of the WHO Malaria Elimination Certification Panel (MECP) visited two border counties, Menglian and Yingjiang, to conduct field assessment for China’s national malaria elimination certification in May 2021. The experts of the WHO MECP highly appreciated the infrastructure and equipment, the competence of the staff of the health system and supporting organization, the data management and the record system during their visits.

**Table 3:** The years of malaria transmission interrupted and malaria free certificated for eight border prefectures, Yunnan

Prefectures	Transmission interrupted	Malaria free certificated
Bordering with Vietnam only		
Honghe (3 counties: Luchun, Jinping, Hekou)^a^	2012	2015
Wenshan (3 counties: Malipo, Maguan, Funing)^a^	2013	2016
Bordering with Vietnam, Lao PDR and Myanmar		
XishuangBanna (3 counties: Menghai, Jinghong, Mengla)^a^	2014	2017
Puer (4 counties: Ximeng, Lancang, Menglian, Jiangcheng)^a^	2016	2018
Bordering with Myanmar only		
Baoshan (2 counties: Tenchong, Longling)^a^	2014	2017
Lincang (3 counties: Zhenkang, Gengma, Cangyuan)^a^	2016	2018
Nujiang (3 counties: Gongshan, Fugong, Lushui)^a^	2016	May 2019
Dehong (4 counties: Yingjiang, Longchuan, Ruili, Mangshi)^a^	2017	September 2019

^a^Bracketed words indicate the numbers and names of border counties

## Discussion

Malaria elimination in the international border areas is one of the challenges that countries face today in their path to malaria elimination. Interruption of malaria transmission and continuous maintenance of malaria free in the Yunnan border area allowed the WHO’s certification of malaria elimination for China [2, 7]. This case study presented the story of malaria from hyperendemicity to elimination in the Yunnan border area. The following experiences and lessons can be learned from this case study.

### Experiences

#### Universal coverage of malaria surveillance

The WHO certification of malaria elimination requires applicant countries to provide evidence that (1) local malaria transmission has been fully interrupted, resulting in zero indigenous human malaria cases for at least the past 3 consecutive years (36 months), and (2) an adequate program for preventing reintroduction of malaria transmission is fully functional throughout the country [17, 28]. The “1-3-7” approach of malaria elimination [24] can only be performed after malaria cases are detected. Finding malaria cases in time is the prerequisite of using the “1-3-7” approach to interrupt and prevent further transmission. To ensure the sensitivity of malaria surveillance, a surveillance system of malaria cases in the border area has gradually achieved universal coverage in the elimination stage, which includes proactive and passive case detection, community-based malaria detection and screening of migrants and travellers in frontier townships. Due to few malaria cases during the elimination stage, malaria diagnosis and treatment can no longer be a money-making channel. Based on the local governmental health policy, private sector, village leaders and village health workers help to monitor migrants and refer febrile patients to perform tests for malaria in health institutions with laboratory test. Remote villages have trained health or malaria workers who can use RDTs to test febrile patients for malaria [10].

#### Accurate and dynamically adjusted stratification

The WHO recommends that stratification should be initially performed at the lowest geographical level for which operational decisions can be made [17]. In the 1990s, Yunnan developed natural village-based stratification to perform cost-effective interventions, and the stratification and intervention measures were adjusted every year [19]. Appropriate investment made it possible to fully carry out natural village-based stratification and interventions from 2003 to 2013. The integrated interventions dramatically reduced the malaria burden. The WHO also recommends that interventions are expected to change the epidemiology of malaria rapidly and profoundly, and the stratification of malaria maps should be revised frequently. As transmission intensity is progressively reduced, stratification needs to include vulnerability and receptivity to malaria [17].

“The Action Plan of China Malaria Elimination (2010–2020)” defined a county as a unit of elimination. The 25 border counties were categorized into two tiers according to the national stratification standards for malaria elimination, namely 17 type I counties and eight type II counties [25, 26]. Yunnan interrupted malaria transmission in 2017, and the national stratification standards for malaria elimination were no longer suitable for the actual situation. Yunnan stratified the 25 border counties into three types (A, B and C) in 2017 and then identified 16 natural villages with high risks of border-spill malaria in 2018 (Additional file 1) to guide resource allocation and the use of a more targeted strategy.

Based on the experiences of malaria control from hyperendemicity to elimination, Yunnan designed the “3 + 1” strategy in 2019 to prevent reintroduction of malaria transmission, namely, (1) comprehensive and intensive malaria interventions in the area within a 2.5 km wide perimeter along the international border to prevent border-spill malaria, (2) community-based malaria surveillance to identify international migrants with possible malaria in the frontier townships, (3) consolidate surveillance into normal health services to maintain vigilance of health personnel to malaria signs, and + 1) emphasize the need to strengthen collaboration with neighboring countries to reduce their malaria burden with a clear focus on border areas with China [10]. The “3 + 1” strategy is in accordance with the principle of the WHO recommended malaria elimination strategy [17].

#### Clearing parasite reservoirs

A comprehensive malaria control strategy includes clearing parasites with antimalarial treatments, interrupting transmission by vector control and protecting vulnerable individuals. Drug-based treatment is the primary intervention in malaria control and elimination, and clearing parasites with antimalarial drugs is the most direct and effective approach. Asymptomatic and submicroscopic parasite density, especially for *P. vivax*, and limitations of microscopist ability and RDTs may lead to underdetection or misdiagnosis [18, 22]. To clear parasite reservoirs for the reduction of malaria infectious sources, expanded clinical and radical cure treatments were conducted in highly endemic years in the border area. The expanded clinical treatment is that the treatment includes both lab confirmed cases and suspected malaria cases in health facilities. The expanded radical cure treatment is that treatment includes people with both history of lab confirmed malaria and suspected malaria in the last 2 years. The ratios of clinical and radical cure treatment to laboratory-confirmed malaria cases were approximately three during 2003‒2006. To accelerate the malaria elimination process, the ratio of radical cure treatment versus laboratory-confirmed malaria cases reached 17.3 in 2010 due to the expanded radical cure treatment (Table 2). Based on these experiences and results of the intervention trial in Cambodia [32], mass drug administration can rapidly reduce the malaria burden in hyperendemic areas; however, it might not be necessary for mesoendemic situations. When malaria endemicity is still high, treatment for all confirmed, clinical and suspected cases, not just targeting confirmed malaria cases, might be necessary [18, 22]. After parasite reservoirs cleared, clinically presumptive treatment of suspected cases is not recommended again. Confirmatory diagnosis for treatment with antimalarial drugs is recommended and practiced because of a few of malaria cases and the high accessibility of laboratory malaria diagnosis for people in the border area. The high accessibility of laboratory test for malaria is assured by the improvement of the laboratory test capacity in public health facilities and the locally improved transportation for residents.

#### Comprehensive interventions

A systematic network literature review compared malaria prevention measures, including ITNs including long lasting insecticidal bed nets and insecticidal-treated bed nets, IRS, prophylactic drugs (PD) and untreated nets (UN), against no intervention. The study demonstrated that only ITN [rate ratio (RR): 0.5, 95% *CI:* 0.3–0.7] showed preventive efficacy precision while other methods, PD (RR: 0.2, 95% *CI:* 0.004–15.4), IRS (RR: 0.6, 95% *CI:* 0.2–1.6) and UN (RR: 0.7, 95% *CI:* 0.3–1.9), indicated considerable uncertainty associated with their point estimates [33]. The results of the review document that no single preventive measures can certainly prevent malaria. An analysis of simulated trial data using a transmission model also documents that a longer duration of prophylaxis leads to a greater measured efficacy of radical cure treatment for *P. vivax*, particularly at higher transmission intensities [34]. The results of this study indicate that integrated interventions are more effective than a single measure.

To control and eliminate malaria, integrated interventions, including proactive and passive case detection, vector surveillance and evidence-based vector control and preventive treatment with drugs, have been used in the border area. In the border area, approximately 100 thousand people received prophylactic drugs for prevention in 2003, and then approximately 2500 people in border communities that neighbouring with the hyperendemic areas of Myanmar received prophylactic drugs to prevent border-spill malaria in 2020. Because of lacking the powerful data on border-spill malaria caused by anopheline mosquitoes infected with malaria parasites, the WHO just recommends prophylactic drugs for travellers in malaria endemic countries, not in the setting of malaria elimination [17, 28]. There is a viewpoint that prophylactic drugs should no longer be used in the phase of malaria elimination in China. However, when vector control measures cannot effectively prevent border-spill malaria, the intervention of prophylactic drugs is still needed for people residing in communities bordering the hyperendemic areas of neighboring countries [10] as well as travellers who want to go to endemic countries [22].

### Lessons

#### Reduced collaboration increased the risk of malaria reintroduction

Communication and collaborative activities were significantly reduced after China’s GFATM malaria project was terminated in 2014. A slight malaria resurgence has appeared in some border areas of Myanmar since 2014 [18, 35]. The number of imported malaria cases correspondingly increased from 358 in 2013 to 594 cases in 2015 in Yunnan. The Laiza and nearby areas of KR2 with a population of approximately 30 thousand persons, are one of the malaria hotspot areas in the border area of Myanmar [10]. The number of reported malaria cases increased from 518 in 2013 to 2367 in 2016. The strengthened collaborative interventions between China and Myanmar during 2017‒2019 reduced the number of malaria cases to 274 in 2019. However, reduced collaborative interventions due to the COVID-19 pandemic led to malaria resurgence again, and a total of 1532 cases were reported in Laiza and nearby areas of KR2 from January to November 2021. The example of Laiza and nearby areas documents that reduced communication and collaboration may increase malaria incidence in the border areas of neighbouring countries and increase the risk of malaria reintroduction in China. In contrast, a reduction in malaria burden in the border area of neighbouring countries can help decrease the threat of malaria importation and reintroduction.

#### Maintaining vigilance of health personnel

Vigilance of health personnel, especially clinical doctors in hospitals, is critical to reduce imported malaria death and prevent reintroduction of malaria transmission in elimination settings [17, 28]. Under the current technical and transportation conditions in China, travelers from malaria-endemic countries can always obtain laboratory tests for malaria in time as long as clinical doctors recognize the necessity of test. In fact, a number of imported malaria deaths are mainly attributable to the delayed diagnosis of malaria because of clinical doctors losing their vigilance. For example, in November 2021, a Burmese patient with kidney failure was hospitalized in a county hospital in the border area. His resident doctor did not recognize the necessity of malaria testing for more than 3 weeks because of the lack of vigilance for malaria. The patient had to be moved to a high-level hospital due to his worsened condition, and then the high-level hospital tested him with *P. malariae*, which was one of the reasons for his kidney failure. Reducing vigilance and technical capacity in malaria diagnosis and treatment due to rarely seeing malaria patients anymore is therefore one of the challenges to prevent the reintroduction of malaria transmission in elimination settings [10].

#### Challenges in the context of the COVID-19 pandemic

The Yunnan border area is also one of the areas facing a high risk of the COVID-19 pandemic in China. To fight the COVID-19 pandemic, some human and financial resources were moved from malaria control to the response to the COVID-19 pandemic. In July 2021, when Yunnan tried to communicate with the Health Authority of Myanmar KR2 to collaborate in rolling back the resurgence of malaria, the KR2 Department of Health responded that they were too busy responding to the COVID-19 pandemic to have human resources fighting malaria. Although the border crossing is strictly limited under the context of the COVID-19 pandemic in Yunnan, the increased malaria incidence in the KR2 has led to malaria spilling over the boundary by *Anopheles* mosquitoes into communities in the Yunnan border area. From January to November 2021, Yingjiang County reported a total of 70 cases, and 63 of them were categorized into border-spill malaria cases. In the context of the COVID-19 pandemic and border collaboration limitations for malaria, comprehensive intervention, including proactive and passive case detection, vector surveillance, evidence-based vector control and preventative treatment with antimalarial drugs, should be undertaken to prevent border-spill malaria within a 2.5 km-wide perimeter along the boundary in Yunnan [10].

## Conclusions

Malaria from hyperendemicity to elimination in the Yunnan border area can be attributed to governmental commitment, comprehensively effective interventions and collaboration with neighbouring countries based on the local context. Although malaria has been eliminated, and reintroduction of malaria transmission has been prevented, malaria importation from the endemic areas of neighbouring countries is still a continuous threat. Comprehensive interventions are continuously essential in preventing the reintroduction of malaria transmission. Access to technical measures requires strong governmental and social support. Other border areas should perform their own intervention trials to develop their own effective strategy of malaria control and elimination in the context of the governing system, malaria burden, health service structure, socioeconomic development and ecology. It can be helpful to refer to and adopt the experiences and lessons from this paper in their own malaria elimination program.

## Supplementary Information

**Additional file 1: Table S1.** Malaria area stratification and interventions in border areas, Yunnan, 2003‒2013. **Table S2.** The annual coverage of laboratory tests for malaria parasites and preventive treatment in the Yunnan border area, 2003‒2020. **Table S3.** The annual parasite incidence (API) in the Yunnan border area, 2003‒2013. **Table S4.** The number of malaria cases detected and the categories in the Yunnan border area, 2014‒2020. **Table S5.** Annual parasite incidence (API) in 25 border counties, Yunnan 2003, 2006, 2010 and 2013. **Table S6.** Malaria cases detected and categories in 25 border counties, Yunnan, 2014‒2020. **Table S7.** High risk villages of imported malaria by parasite-infected anophelines in 2018.

## Data Availability

Not applicable.

## Abbreviations

API: Annual parasite incidence
WHO: World Health Organization
GMS: Greater Mekong Subregion
COVID-19: Coronavirus disease 2019
YIPD: Yunnan Institute of Parasitic Diseases
CISDCP: Chinese Information System for Disease Control and Prevention
RDTs: Rapid diagnostic tests
IRS: Indoor residual spraying
GFATM: Global Fund to Fight AIDS, Tuberculosis and Malaria
CDC: Centre for Disease Control and Prevention
KR2: Kachin Special Region II
MECP: Malaria Elimination Certification Panel
ITNs: Insecticide treated bed nets
RR: Rate ratio
