# Supplementary material for: Malaria from hyperendemicity to elimination along international borders in Yunnan, China during 2003‒2020: a case study
Source: Infect Dis Poverty. 2022 May 10;11:51. doi: 10.1186/s40249-022-00972-2 (PMC9088148; doi:10.1186/s40249-022-00972-2)
Supplement: Supplementary file 1 — Additional file 1: Table S1. Malaria area stratification and interventions in border areas, Yunnan, 2003‒2013. Table S2. The annual coverage of laboratory tests for malaria parasites and preventive treatment in the Yunnan border area, 2003‒2020. Table S3. The annual parasite incidence (API) in the Yunnan border area, 2003‒2013. Table S4. The number of malaria cases detected and the categories in the Yunnan border area, 2014‒2020. Table S5. Annual parasite incidence (API) in 25 border counties, Yunnan 2003, 2006, 2010 and 2013. Table S6. Malaria cases detected and categories in 25 border counties, Yunnan, 2014‒2020. Table S7. High risk villages of imported malaria by parasite-infected anophelines in 2018. [file 40249_2022_972_MOESM1_ESM.docx]

**Additional file**

**Table S1. Malaria area stratification and interventions in border areas, Yunnan, 2003-2013**

| Tier | definition | Interventions |
| --- | --- | --- |
| Type 1 | A village API ≥ 1%, or malaria clinical attack rate ≥ 10% last year | 1. Mass drug administration for radical cure and preventive treatment; 2. Presumptive treatment of all febrile patients for malaria; 3. Two rounds of indoor residual spraying (IRS) or dipping bed nets with pyrethroid insecticides. |
| Type 2 | A village API < 1%, or malaria clinical attack rate < 10% last year, but there are indigenous cases in the last 3 years. | 1. Radical cure treatment of people with malaria attack history in last 2 years; 2. Presumptive treatment of suspected malaria cases and febrile patients without clear causes for malaria 3. One rounds of IRS or dipping bed nets with pyrethroid insecticides. |
| Type 3 | A village without indigenous cases, only imported cases in the last 3 years | 1. Radical cure treatment of people with malaria attack history in last 2 years; 2. Presumptive treatment of suspected malaria cases and febrile patients without clear causes for malaria; 3. IRS with pyrethroid insecticides to malaria patient’s and neighbouring houses |
| Type 4 | A village without any malaria cases in the last 3 years |  |

**Table S2. The annual coverage of laboratory tests for malaria parasites and preventive treatment in the Yunnan border area, 2003‒2020**

| Year | Population | No. tested | Test rate (%) | No. persons with prophylaxes | Prophylaxis coverage (%) |
| --- | --- | --- | --- | --- | --- |
| 2003 | 6,051,430 | 224,486 | 3.71 | 93,051 | 1.54 |
| 2004 | 6,112,925 | 236,099 | 3.86 | 89,815 | 1.47 |
| 2005 | 6,167,180 | 191,964 | 3.11 | 62,803 | 1.02 |
| 2006 | 6,400,789 | 214,958 | 3.36 | 65,443 | 1.02 |
| 2007 | 6,634,400 | 219,068 | 3.30 | 65,787 | 0.99 |
| 2008 | 6,868,010 | 214,320 | 3.12 | 60,087 | 0.87 |
| 2009 | 6,874,053 | 212,763 | 3.10 | 55,521 | 0.81 |
| 2010 | 6,934,526 | 213,037 | 3.07 | 55,819 | 0.80 |
| 2011 | 7,178,704 | 193,607 | 2.70 | 44,179 | 0.62 |
| 2012 | 7,431,481 | 202,409 | 2.72 | 35,521 | 0.48 |
| 2013 | 7,702,707 | 184,740 | 2.40 | 47,833 | 0.62 |
| 2014 | 7,973,935 | 165,605 | 2.08 | 23,339 | 0.29 |
| 2015 | 8,044,084 | 172,797 | 2.15 | 16,512 | 0.21 |
| 2016 | 8,114,851 | 158,496 | 1.95 | 9,798 | 0.12 |
| 2017 | 8,186,240 | 150,479 | 1.84 | 12,166 | 0.15 |
| 2018 | 8,485,013 | 126,807 | 1.49 | 8,499 | 0.10 |
| 2019 | 8,783,787 | 106,101 | 1.21 | 1,865 | 0.02 |
| 2020 | 9,093,082 | 91,581 | 1.01 | 2,506 | 0.03 |

**Table S3. The annual parasite incidence (API) in the Yunnan border area, 2003‒2013**

| Year | Population | No. cases | API / 10,000 person-years |
| --- | --- | --- | --- |
| 2003 | 6,051,430 | 10,349 | 17.10 |
| 2004 | 6,112,925 | 8,515 | 13.93 |
| 2005 | 6,167,180 | 9,538 | 15.47 |
| 2006 | 6,400,789 | 8,666 | 13.54 |
| 2007 | 6,634,400 | 4,872 | 7.34 |
| 2008 | 6,868,010 | 2,762 | 4.02 |
| 2009 | 6,874,053 | 2,045 | 2.97 |
| 2010 | 6,934,526 | 1,570 | 2.26 |
| 2011 | 7,178,704 | 850 | 1.18 |
| 2012 | 7,431,481 | 449 | 0.60 |
| 2013 | 7,702,707 | 475 | 0.62 |

**Table S4．The number of malaria cases detected and the categories in the Yunnan border area, 2014‒2020**

| Year | 2014 | 2015 | 2016 | 2017 | 2018 | 2019 | 2020 | Total |
| --- | --- | --- | --- | --- | --- | --- | --- | --- |
| *Plasmodium* species |  |  |  |  |  |  |  |  |
| *P. falciparum* (*Pf*) | 60 | 48 | 20 | 11 | 10 | 2 | 2 | 153 |
| *P. vivax* (*Pv*) | 331 | 428 | 297 | 251 | 155 | 147 | 133 | 1,742 |
| *P. malariae* | 1 | 1 | 0 | 0 | 1 | 0 | 0 | 3 |
| Mixed (*Pf* + *Pv*) | 1 | 1 | 1 | 0 | 0 | 0 | 2 | 5 |
| Category |  |  |  |  |  |  |  |  |
| Indigenous cases | 33 | 11 | 1* | 0 | 0 | 0 | 0 | 45 |
| Imported cases | 360 | 467 | 317 | 264 | 169 | 149 | 137 | 1,863 |
| Total | 393 | 478 | 318 | 264 | 169 | 149 | 137 | 1,908 |

**Note**: *The last indigenous case (*P. vivax*) occurred in Yingjiang County on the China-Myanmar border in April 2016. It is also the last indigenous case in China.

**Table S5. Annual parasite incidence (API) in 25 border counties, Yunnan 2003, 2006, 2010 and 2013**

| County | 2003 | | | 2006 | | | 2010 | | | 2013 | | |
| --- | --- | --- | --- | --- | --- | --- | --- | --- | --- | --- | --- | --- |
|  | **Population** | **No. cases** | **API (/10,000)** | **Population** | **No. cases** | **API (/10,000)** | **Population** | **No. cases** | **API (/10,000)** | **Population** | **No. cases** | **API (/10,000)** |
| Tenchong | 585,587 | 1,670 | 28.52 | 619,394 | 2,206 | 35.62 | 671,043 | 556 | 8.29 | 745,378 | 149 | 2.00 |
| Longling | 243,539 | 547 | 22.46 | 257,598 | 568 | 22.05 | 279,078 | 76 | 2.72 | 309,994 | 14 | 0.45 |
| Jiangcheng | 82,092 | 52 | 6.33 | 86,831 | 19 | 2.19 | 94,071 | 6 | 0.64 | 104,492 | 5 | 0.48 |
| Menglian | 100,334 | 278 | 27.71 | 106,127 | 84 | 7.92 | 114,976 | 2 | 0.17 | 127,713 | 9 | 0.70 |
| Lancang | 411,370 | 109 | 2.65 | 435,119 | 56 | 1.29 | 471,402 | 20 | 0.42 | 523,623 | 4 | 0.08 |
| Ximeng | 72,970 | 555 | 76.06 | 77,183 | 220 | 28.50 | 83,619 | 14 | 1.67 | 92,882 | 2 | 0.22 |
| Zhenkang | 158,072 | 490 | 31.00 | 167,198 | 191 | 11.42 | 181,140 | 15 | 0.83 | 201,206 | 3 | 0.15 |
| Gengma | 285,679 | 351 | 12.29 | 302,172 | 163 | 5.39 | 327,368 | 18 | 0.55 | 363,633 | 11 | 0.30 |
| Cangyuan | 155,062 | 298 | 19.22 | 164,014 | 326 | 19.88 | 177,690 | 96 | 5.40 | 197,374 | 6 | 0.30 |
| Jinping | 261,781 | 240 | 9.17 | 276,894 | 247 | 8.92 | 299,983 | 23 | 0.77 | 333,214 | 0 | 0.00 |
| Luchun | 186,987 | 54 | 2.89 | 197,782 | 48 | 2.43 | 214,274 | 22 | 1.03 | 238,010 | 0 | 0.00 |
| Hekou | 870,171 | 89 | 1.02 | 920,408 | 124 | 1.35 | 997,157 | 18 | 0.18 | 1,107,618 | 1 | 0.01 |
| Malipo | 246,275 | 78 | 3.17 | 260,493 | 109 | 4.18 | 282,214 | 3 | 0.11 | 313,477 | 0 | 0.00 |
| Maguan | 319,245 | 132 | 4.13 | 337,676 | 103 | 3.05 | 365,833 | 30 | 0.82 | 406,359 | 2 | 0.05 |
| Funing | 355,730 | 112 | 3.15 | 376,267 | 25 | 0.66 | 407,643 | 0 | 0.00 | 452,800 | 0 | 0.00 |
| Jinghong | 401,337 | 1,168 | 29.10 | 424,507 | 217 | 5.11 | 459,905 | 10 | 0.22 | 510,851 | 5 | 0.10 |
| Menghai | 273,639 | 145 | 5.30 | 289,436 | 62 | 2.14 | 313,571 | 6 | 0.19 | 348,308 | 1 | 0.03 |
| Mengla | 182,426 | 510 | 27.96 | 192,958 | 178 | 9.22 | 209,048 | 14 | 0.67 | 232,205 | 8 | 0.34 |
| Ruili | 150,501 | 652 | 43.32 | 159,190 | 726 | 45.61 | 172,464 | 115 | 6.67 | 191,569 | 133 | 6.94 |
| Luxi | 54,728 | 1,138 | 207.94 | 57,887 | 873 | 150.81 | 62,714 | 50 | 7.97 | 69,662 | 25 | 3.59 |
| Yingjiang | 237,062 | 917 | 38.68 | 250,748 | 1,368 | 54.56 | 271,657 | 364 | 13.40 | 301,751 | 72 | 2.39 |
| Longchuan | 164,183 | 645 | 39.29 | 173,662 | 529 | 30.46 | 188,143 | 84 | 4.46 | 208,985 | 14 | 0.67 |
| Lushui | 141,380 | 21 | 1.49 | 149,542 | 34 | 2.27 | 162,012 | 14 | 0.86 | 179,959 | 6 | 0.33 |
| Fugong | 80,267 | 31 | 3.86 | 84,901 | 146 | 17.20 | 91,981 | 9 | 0.98 | 102,170 | 4 | 0.39 |
| Gongshan | 31,012 | 67 | 21.60 | 32,803 | 44 | 13.41 | 35,538 | 5 | 1.41 | 39,475 | 1 | 0.25 |
| Total Indigenous | 6,051,430 |  | 0.00 | 6,400,789 |  | 0.00 | 6,934,525 | 502 | 0.72 | 7,702,707 | 49 | 0.06 |
| Total imported | - | - | - | - |  | - | - | 1,068 | - | - | 426 | - |
| Overall | **6,051,430** | **10,349** | **17.10** | **6,400,789** | **8,666** | **13.54** | **6,934,525** | **1,570** | **2.26** | **7,702,707** | **475** | **0.62** |

**Note:** Malaria cases were not categorized into indigenous and imported cases in 2003 and 2006.

**Table S6. Malaria cases detected and categories in 25 border counties, Yunnan, 2014-2020**

|  | 2014 | 2015 | 2016 | Subtotal | 2017 | 2018 | 2019 | 2020 | Subtotal | Total |
| --- | --- | --- | --- | --- | --- | --- | --- | --- | --- | --- |
| Total | 393 | 478 | 318 | 1,189 | 264 | 169 | 149 | 137 | 719 | 1,908 |
| *Plasmodium* species |  |  |  |  |  |  |  |  |  |  |
| *P. falciparum* (*Pf*) | 60 | 48 | 20 | 128 | 11 | 10 | 2 | 2 | 25 | 153 |
| *P. vivax* (*Pv*) | 331 | 428 | 297 | 1,056 | 251 | 155 | 147 | 133 | 686 | 1,742 |
| *P. malariae* | 1 | 1 | 0 | 2 | 0 | 1 | 0 | 0 | 1 | 3 |
| Mixed (*Pf* +*Pv*) | 1 | 1 | 1 | 3 | 0 | 0 | 0 | 2 | 2 | 5 |
| Category |  |  |  |  |  |  |  |  |  |  |
| Indigenous cases | 33 | 11 | 1 (Yingjiang) | 45 | 0 | 0 | 0 | 0 | 0 | 45 |
| Imported cases | 360 | 467 | 317 | 1,144 | 264 | 169 | 149 | 137 | 719 | 1,863 |
| County |  |  |  |  |  |  |  |  |  |  |
| Yingjiang | 87 | 175 | 186 | 448 | 179 | 106 | 92 | 75 | 452 | 900 |
| Tenchong | 122 | 131 | 55 | 308 | 28 | 18 | 22 | 23 | 91 | 399 |
| Ruili | 106 | 74 | 23 | 193 | 15 | 12 | 8 | 3 | 38 | 231 |
| Mangshi | 25 | 17 | 11 | 53 | 13 | 8 | 13 | 7 | 41 | 94 |
| Longling | 15 | 28 | 7 | 60 | 5 | 5 |  | 1 | 11 | 71 |
| Longchuan | 10 | 7 | 9 | 26 | 17 | 8 | 4 | 16 | 45 | 71 |
| Cangyuan | 8 | 8 | 4 | 20 | 3 | 1 | 5 | 8 | 17 | 37 |
| Mengla | 3 | 14 | 3 | 20 | 1 | 1 | 0 | 0 | 2 | 22 |
| Gengma | 0 | 3 | 4 | 7 | 2 | 2 | 3 | 2 | 9 | 16 |
| Menglian | 3 | 4 | 3 | 10 | 0 | 1 | 0 | 0 | 1 | 11 |
| Jinghong | 1 | 5 | 2 | 8 | 0 | 1 | 0 | 0 | 1 | 9 |
| Lushui | 1 | 6 | 1 | 8 | 0 | 0 | 0 | 0 | 0 | 8 |
| Jinping | 0 | 0 | 0 | 0 | 1 | 2 | 1 | 2 | 6 | 6 |
| Fugong | 2 | 3 | 1 | 6 | 0 | 0 | 0 | 0 | 0 | 6 |
| Lancang | 1 | 1 | 3 | 5 | 0 | 0 | 0 | 0 | 0 | 5 |
| Jiangcheng | 1 | 1 | 2 | 4 | 0 | 0 | 0 | 0 | 0 | 4 |
| Gongshan | 4 | 0 | 0 | 4 | 0 | 0 | 0 | 0 | 0 | 4 |
| Ximeng | 3 | 0 | 0 | 3 | 0 | 0 | 0 | 0 | 0 | 3 |
| Maguan | 1 | 0 | 1 | 2 | 0 | 1 | 0 | 0 | 1 | 3 |
| Zhenkang | 0 | 1 | 0 | 1 | 0 | 1 | 0 | 0 | 1 | 2 |
| Luchun | 0 | 0 | 2 | 2 | 0 | 0 | 0 | 0 | 0 | 2 |
| Funing | 0 | 0 | 1 | 1 | 0 | 1 | 0 | 0 | 1 | 2 |
| Menghai | 0 | 0 | 0 | 0 | 0 | 1 | 1 | 0 | 2 | 2 |
| Hekou | 0 | 0 | 0 | 0 | 0 | 0 | 0 | 0 | 0 | 0 |
| Malipo | 0 | 0 | 0 | 0 | 0 | 0 | 0 | 0 | 0 | 0 |

**Table S7. High risk villages of imported malaria by parasite-infected anophelines in 2018**

| No. | Natural village name | Township or town | County |
| --- | --- | --- | --- |
| 1 | Daoshui village team 4 | Nansan | Zhenkang |
| 2 | Yakou village | Mengdui |  |
| 3 | Mengding Plantation division team 2 | Mengding | Genma |
| 4 | Hanhong Mangbeng Village team 1 |  |  |
| 5 | Manghai new market | Manghai | Mangshi |
| 6 | Xiabanlao village Longyaoshan | Banlao | Cangyuan |
| 7 | Nabang village qiaotou | Nabang | Yingjiang |
| 8 | Nabang village Xinguomen |  |  |
| 9 | Nabang village Lisu village |  |  |
| 10 | Nabang village Jingpo village |  |  |
| 11 | Nabang village Kayahe |  |  |
| 12 | Nabang village nabang port |  |  |
| 13 | Street village vegetable team |  |  |
| 14 | Daolong village |  |  |
| 15 | Caobajie Dongpengyang | Kachang |  |
| 16 | Longpen village | Taiping |  |
